# Uncovering battery electrochemical mechanisms by artificial intelligence

**DOI:** 10.1093/nsr/nwaf442

**Published:** 2025-10-16

**Authors:** Zhiyuan Han, Jiaqi Zhou, Gongxun Lu, Zhihong Piao, Shengyu Tao, Runhua Gao, Chuang Li, Xuan Zhang, Guangmin Zhou

**Affiliations:** Tsinghua Shenzhen International Graduate School, Tsinghua University, Shenzhen 518055, China; Tsinghua Shenzhen International Graduate School, Tsinghua University, Shenzhen 518055, China; Tsinghua Shenzhen International Graduate School, Tsinghua University, Shenzhen 518055, China; Tsinghua Shenzhen International Graduate School, Tsinghua University, Shenzhen 518055, China; Tsinghua Shenzhen International Graduate School, Tsinghua University, Shenzhen 518055, China; Tsinghua Shenzhen International Graduate School, Tsinghua University, Shenzhen 518055, China; Tsinghua Shenzhen International Graduate School, Tsinghua University, Shenzhen 518055, China; Tsinghua Shenzhen International Graduate School, Tsinghua University, Shenzhen 518055, China; Tsinghua Shenzhen International Graduate School, Tsinghua University, Shenzhen 518055, China

**Keywords:** electrochemistry, battery, AI for battery

## Abstract

Batteries have been driving the sustainable energy transition by empowering critical applications such as consumer electronics, electric vehicles and grid energy storage systems. Key challenges in battery research and development require a fundamental understanding of the dynamic evolution of electrochemical interfaces, cross-dimensional and cross-scale relationships, and intertwined interaction electrochemical processes. Advanced characterization and theoretical computation-based methods generate considerably discrete, heterogeneous and condition-sensitive but huge data streams. Such complexity leads to difficulties in human expert-oriented interpretations. Artificial intelligence (AI) offers new promise for handling this gigantic amount of data by enabling efficient curation, preprocessing, model construction, deployment, optimization and, most importantly, interpretation. While AI integration into battery research has been well documented, this Review pays special attention to its potential to uncover three critical yet outstanding chemical mechanistic aspects. First, AI reveals temporal evolution mechanisms by denoising and statistically analyzing large, uneven-quality time-resolved data. Second, it discovers latent relationships across data with multiple dimensions and scales, which are difficult to infer from established theories alone. Third, it decouples complex interaction networks by identifying dominating factors and their relative contributions. We highlight the importance of standardized data collection, open-source data deposition, domain expert knowledge integration, application of advanced AI models, and experiment optimization to scalable and electrochemistry-informed AI applications. While emerging tools like large language models and autonomous agents hold promise, their impact will rely on thoughtful human–AI collaboration that preserves safety, ethics and mechanistic insight.

## Teaser

This Review centers on how AI can uncover fundamental electrochemical mechanisms in batteries through three critical yet unresolved aspects—tracking temporal evolution, discovering cross-dimensional mechanisms, and decoupling complex interaction networks.

## INTRODUCTION

**Figure 1.**
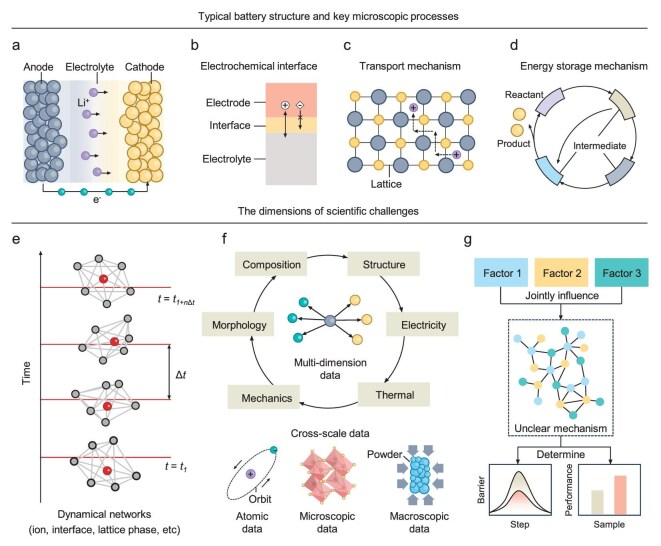
Representative structure, processes and emerging challenges in battery research. (a) Simplified schematic of a typical battery, showing ion transport and electron flow between anode, electrolyte and cathode. (b–d) Key microscopic processes: ion and electron transfer across the electrochemical interface (b), transport processes within the lattice (c) and energy storage through reaction intermediates and redox cycling (d). (e–g) Emerging scientific challenge: dynamic evolution of entities (red circle) and their interactions with the surrounding network (gray circle) over time (e), complex interplay of cross-dimensional and cross-scale data (f) and decouple the joint effects of multiple interdependent factors on performance (g).

Lithium-ion batteries convert chemical energy and electrical energy through electrochemical reactions (Fig. 1a). They play a pivotal role in accelerating the global transition to decarbonization by advancing renewable energy, popularizing electric vehicles and supporting distributed energy systems. Alongside lithium-ion batteries, a variety of next-generation systems, such as sodium-ion batteries [1], lithium-metal batteries [2] and all-solid-state batteries [3], are actively being developed to address limitations in cost, safety and performance. Across these technologies, the development goals of the battery research community include improving energy and power density [4], extending cycle life [5], enhancing safety [6] and reducing manufacturing costs [7]. Achieving these objectives requires a deeper understanding of the electrochemical mechanism within the battery. Key aspects include electrochemical interface reactions and their stabilization mechanisms [8–10], the coupling transport of various entities such as electrons, ions and solvated complexes [11], and the energy storage mechanisms of next-generation electrode materials [12–14] (Fig. 1b–d). Based on these typical topics of electrochemical mechanisms, three dimensions have emerged in recent battery research. The first dimension is investigating the dynamic evolution of particles and interfaces during battery operation by advanced electrochemical *in situ* characterization (Fig. 1e) [15,16]. This includes charge transfer, ion transport, phase transitions and electrode degradation. Secondly, understanding complex interactions among multidimensional (morphology, structure, composition, electrical, mechanical and thermal properties) and multiscale data (from atomic structures to macroscopic battery performance) has become key (Fig. 1f) [17]. Thirdly, efforts are increasingly directed at decoupling intricate electrochemical processes to reveal the roles and interactions of individual factors, such as thermodynamics, kinetics and mechanical stress (Fig. 1g) [18].

However, the inevitable high noise of *in situ* characterization with ultrahigh temporal resolution limits its ability to fully capture dynamic processes, while manually identifying microscopic phenomena from vast dynamic datasets is both time-consuming and labor-intensive. Integrating multidimensional data, such as composition, structure and morphology, across scales from atomic to macroscopic levels into a unified framework, essential for analyzing non-linear relationships, remains a significant challenge. Additionally, key factors in complex electrochemical processes are often intertwined by many potential variables, making it difficult to determine their relative importance. Compared to traditional methods that rely on hypotheses and predefined assumptions, AI (see key terms in Box 1) methods are data-driven, capable of processing large and noisy datasets, and can learn more complex relationships among many variables. Moreover, AI enables exploration of broader and high-dimensional spaces (Table 1). Therefore, it provides a promising solution to the above challenges [19], helping researchers to statistically analyze vast and uneven-quality data, discovering cross-dimensional relationships, and decoupling dominating factors in complex interaction networks.

**Table 1.** Comparison of traditional methods and AI methods in electrochemical analysis.

Dimension	Traditional method	AI method
Workflow logic	Hypothesis-driven, limited by current domain knowledge	Data-driven, optionally constrained by chemical laws
Data handling	Manual processing, small datasets	Automated processing large datasets
Data quality requirement	High	Mitigate noise through large-scale data
Exploration capability	Limited space	High-dimensional and broad space
Variable relationship	Limited variable numbers, simple relationship	More variable numbers, complex relationship

Box 1. **Key terms in AI for electrochemical research**

1. Feature: a measurable property or characteristic of data, serving as the foundation for analysis and predictions in AI models. Features include physical attributes (e.g. particle size, composition), spectroscopic data, image pixel intensities etc.
2. Feature engineering: the process of transforming raw data into meaningful inputs, either expert knowledge or purely statistically driven to enhance AI model performance.
3. Model: a mathematical representation used by AI to learn patterns and relationships between input features and outputs. It serves as the core mechanism for making predictions and classifications, or uncovering insights.
4. Training set, validation set and testing set: the training set is used to teach the model by identifying patterns and relationships in the data. During training, the validation set is employed to fine-tune hyperparameters and assess the model’s ability to generalize. The testing set, kept entirely separate from training, evaluates the performance of unseen data, ensuring its accuracy and reliability in real-world applications.
5. Supervised learning: trains AI models on labeled data, mapping inputs to known outputs. Once trained, the model predicts outcomes for new data. A typical example of supervised learning is predicting the electrode potential of new solvents based on a labeled dataset of solvents with known electrode potentials.
6. Unsupervised learning: analyzes unlabeled data to uncover hidden patterns or groupings. A typical example is clustering unlabeled XANES spectra to identify distinct atomic configurations.
7. Attention: enables models to dynamically focus on the most relevant parts of the input by assigning different weights to each element, improving task-specific performance.
8. Encoder: a neural network module that transforms input data into a compact representation called an embedding, capturing essential features for downstream tasks.

Previous reviews have primarily focused on AI applications in materials discovery [20–23], multiscale modeling [20,24–26], experimental planning [20,27,28], synthesis optimization [20,28,29], battery manufacturing [20,30], structural characterization [20,29,31], performance diagnosis [20,22,32–34] and recycling strategies [20]. However, despite the growing interest in AI across the battery community, few reviews have systematically examined how AI can be used to address fundamental mechanistic challenges in battery electrochemistry. This gap has limited the field’s ability to fully realize AI’s potential in guiding scientific understanding, not just accelerating workflows. Therefore, this review centers on how AI can uncover fundamental electrochemical mechanisms in batteries. We firstly introduce the AI workflow and methods for battery research, then organize the discussion around three mechanistic challenges: (i) tracking temporal evolution, such as ion transport, structural phase transitions and electrode transformations; (ii) identifying cross-dimensional mechanisms via multimodal data integration, with examples from solvation, electrode and catalytic chemistry; and (iii) decoupling complex interactions in multicomponent systems, including composite architectures, aging behaviors and catalytic networks. The review concludes with a discussion of current limitations and future directions for data-driven mechanistic understanding in electrochemical systems.

## AI METHODS FOR BATTERY RESEARCH

**Figure 2.**
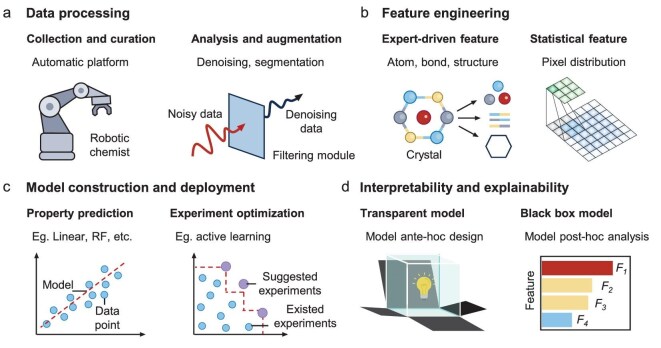
Core stages of AI workflows in advancing battery research. (a) Data processing, including automated data collection and curation via robotic chemists, as well as analysis and augmentation such as denoising and segmentation of noisy data. (b) Feature engineering, including expert-driven features (e.g. atom, bond and structure features) and statistical features (e.g. pixel value distribution and local patterns in image data). (c) Model construction and deployment tasks, including two major applications: property prediction using methods such as linear regression or random forests, and experiment optimization using active learning to prioritize the most informative experiments. (d) Interpretability and explainability address how models can be understood and trusted. Transparent models are interpretable by design, while black-box models require *post hoc* techniques (e.g. feature importance analysis) to enhance the scientific reliability of AI-driven results.

Uncovering electrochemical mechanisms with AI typically contains four key steps: data processing; feature engineering; model construction and deployment; and result interpretation and explanation (Fig. 2). These stages form a unified workflow that bridges raw experimental data and mechanistic understanding.

### Data processing

AI can not only automate data acquisition and information collecting but also assist in organizing, maintaining and optimizing data throughout the whole ongoing process, particularly in high-throughput experiments and real-time measurements. For example, robotic chemists, which refer to AI-powered robotic systems, can rapidly screen materials or systematically acquire imaging, spectroscopic and electrochemical data with minimal human intervention. This improves efficiency and reduces experimental bias. In addition, experimental data, especially from *in situ* techniques, often suffers from noise due to limited resolution or prolonged acquisition times. AI methods can denoise and enhance signal quality. To facilitate further information extraction, data augmentation, such as image segmentation, is often a necessary step before proceeding with further global and local analysis.

### Feature engineering

Feature engineering serves as the critical step in translating raw data into meaningful representations that models can understand. Expert-driven features are extracted from high-dimensional data using domain knowledge. Key examples include atomic properties (e.g. radius, electronegativity, charge density), bond strength and crystal structure, all of which are essential for describing electrode materials and linking AI predictions to electrochemical mechanisms.

Statistical features are derived from data using mathematical methods, independent of domain-specific knowledge. For instance, in image analysis, pixel value distributions capture brightness and contrast, while local patterns reveal texture and spatial relationships. These features complement expert-driven descriptors, enabling AI to uncover trends and anomalies in complex datasets. Combining these two types of features enables both interpretability and flexibility, allowing models to capture subtle trends and generalize across complex datasets.

### Model construction and deployment

Once features are defined, AI models, ranging from traditional machine learning to deep neural networks, can be trained to predict electrochemical properties or guide experiment design.

Using models trained on experimental and simulated datasets, AI can potentially predict electrochemical performance without the need for experiments. This includes metrics such as Li electrode potential, catalytic activity and thermodynamic stability. These predictions help prioritize promising candidates for experimental validation and mechanism analysis.

In addition, AI accelerates the design and optimization of experiments through techniques like Bayesian optimization and active learning. By identifying the most informative experiments to perform, AI minimizes resource consumption and enables targeted exploration of material and process spaces.

### Interpretability and explainability

AI can also help with interpretability and explainability of models [35] to provide mechanistic insights and improve the scientific reliability of discoveries. Interpretability often focuses on model *ante hoc* design, constructing a ‘glass box’ model directly, where the internal logic and decision-making process are inherently understandable.

Explainability involves *post hoc* methods to evaluate the outcomes of a ‘black box’ model. Techniques such as feature importance ranking or Shapley additive explanations (SHAP) help researchers reason through complex model predictions and identify potential mechanisms.

While these four steps outline a general pipeline for applying AI to battery research, their successful implementation depends critically on understanding the models themselves. To assist in better understanding the different AI models, Box 2 provides clear explanations of the methods and terms mentioned in the manuscript, along with typical usage scenarios, workflows and key considerations. Building on this foundation, the following sections present three representative perspectives to illustrate how AI can uncover critical mechanistic questions in battery research.

Box 2. **AI methods and terms mentioned in the manuscript**

1. Sparse coding
  Sparse coding improves low-quality spectroscopic images, like EELS maps of lithium diffusion, by referencing typical patterns from high-quality data. It reconstructs images by combining similar features from a learned pattern set. This method requires representative high-quality data, assumes consistent structures, and may miss rare features.
2. Convolutional neural networks (CNNs)
  CNNs can analyze electrochemical images (e.g. tomography, STEM) to identify patterns like electrode structures, lithium phases or defects. They process images to produce segmentation or classification results. CNNs require large, labeled datasets, are computationally intensive, and may struggle to generalize across different materials or imaging technology settings.
3. Shapley additive explanations (SHAP)
  SHAP is used to interpret machine learning predictions by quantifying how much each feature contributes to outcomes. It helps identify dominant factors. Though powerful, SHAP is computationally demanding and may be less accurate when features are strongly interdependent.
4. Random forest
  Random forest is a widely used model for predicting battery properties (e.g. capacity fade) from features like elemental ratios or particle shapes. It builds decision trees and averages their outputs to improve accuracy. It also helps identify which features matter most but may overfit or become slow with large or complex datasets. Interpretability often requires tools like SHAP.
5. Principal component analysis (PCA)
  PCA can reduce the complexity of large electrochemical datasets by identifying a few main patterns that capture most of the variation. However, PCA assumes linear relationships, may miss rare but important features, and its results depend on how many components are chosen.
6. Physics-constrained networks
  These models integrate physical principles into their training process to guide predictions, potentially helping improve the accuracy of generated electrochemical states. They are especially useful when direct observation is limited. However, success depends on clearly defined physical rules and may require substantial preprocessing, like accurate segmentation of electrode phases.
7. Linear regression and partial least squares regression (PLSR)
  Linear regression and PLSR can model the relationship between electrochemical features (e.g. porosity, molecular structure) and performance metrics (e.g. capacity, voltage). Linear regression is simple and interpretable but limited to low-dimensional, linear patterns. PLSR projects high-dimensional data into latent variables, better handling collinearity. Both require careful preprocessing and assume linear trends, limiting their use for complex or non-linear systems.
8. Generative models
  Generative models can be used to generate new candidate materials (e.g. electrolytes, electrodes) by learning patterns in known data. Models like variational autoencoders or diffusion models can create chemically valid outputs, sometimes guided by optimization. Limitations include synthetic infeasibility, data requirements and the need for additional validation (e.g. DFT or experiments).
9. Graph-based models
  Graph-based models can be used to analyze electrochemical systems by representing atoms or molecules as nodes and their interactions as edges (e.g. bonds, distances). These models can predict properties like binding energy or redox potential based on graph structure and node features. Challenges include high data requirements, sensitivity to graph construction and limited interpretability.
10. Transfer learning
  Transfer learning can boost performance on small electrochemical datasets by adapting models pretrained on large, related data. It reuses learned features and fine-tunes them for specific tasks like rare electrolyte screening. It works best when source and target domains are similar, but may fail with domain mismatch. Meanwhile, pretraining is costly, and interpretability is limited.
11. Reinforcement learning
  Reinforcement learning can be used to optimize decision-making in sequential electrochemical tasks, such as discovering stable synthesis routes. The model learns by trial and error through feedback from simulated or experimental environments. It requires well-designed reward strategies and extensive training iterations. High computational cost and limited interpretability remain key challenges.
12. Active learning and Bayesian optimization
  These methods can accelerate discovery by selecting the most informative experiments, such as testing uncertain battery materials. Active learning reduces labeling needs, while Bayesian optimization builds a predictive model to guide sampling. Both are useful when experiments are costly but depend on accurate uncertainty estimates.

## AI REVEALING NEW MECHANISMS BY TRACKING TEMPORAL EVOLUTION

Recent advances in *in situ* characterization techniques, including *in situ* X-ray fluorescence microscopy [15], optical scattering microscopy [36], Raman spectroscopy [37], transmission electron microscopy (TEM) [38–40], and UV–visible (UV–vis) spectroscopy [41], have enabled a shift from static to time-resolved observations of electrochemical systems. This progress allows researchers to investigate the temporal evolution of electrochemical systems, such as atomic dynamics at electrified solid–liquid interfaces [42], collective reactions at sulfur electrode interfaces [40] and heterogeneous reaction kinetics within electrode particles [18].

The promise of such techniques lies in their ability to reveal dynamic behaviors that are inaccessible from endpoint measurements. However, practical limitations remain. High temporal resolution often comes at the cost of signal-to-noise ratio, resulting in large datasets that are time-sequenced with causal dependencies, unstructured in format (e.g. image data) and difficult to annotate manually. Identifying subtle and transient evolution is particularly difficult without computational assistance.

**Figure 3.**
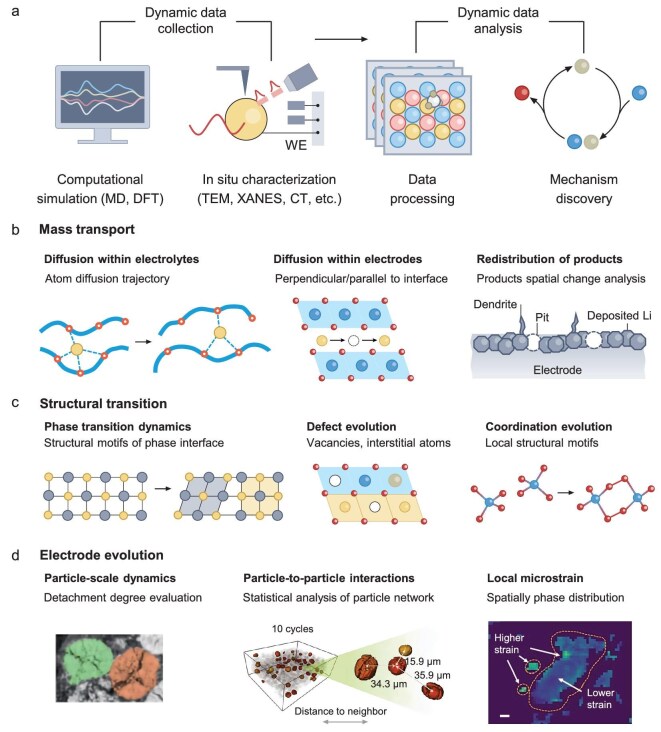
AI revealing mechanism by tracking temporal evolution. (a) Traditional workflow for tracking temporal evolution, encompassing dynamic data collection via computational simulations and (quasi-) *in situ* characterization, followed by data processing and interpretation to uncover evolution mechanisms. (b) Mass transport processes, including ion diffusion within electrolytes (left), ion diffusion in electrode lattices (center) and spatial redistribution of reaction products such as dendrite formation or Li plating (right). (c) Structural transitions, encompassing phase transition dynamics (left), defect evolution (center) and changes in coordination structures (right). (d) Electrode evolution, captured by AI-augmented image analysis, includes particle-scale dynamics (left), changes in particle-to-particle interactions across cycling (center) and local mechanical strain distributions (right). Scale bar, 10 μm. Part (d) adapted from ref. [77], Springer Nature Limited; ref. [79], Science (AAAS); ref. [81], Wiley-VCH GmbH.

AI has thus emerged as a tool to support the analysis of time-series characterization data by automating noise reduction, feature extraction and statistical analysis (Fig. 3a). Beyond using AI only for denoising or pattern recognition, a more critical goal is to identify patterns in time-series data that may reflect chemically meaningful dynamics. However, this remains challenging due to the lack of annotated datasets with ground-truth chemical information and the difficulty of defining proper task labels for chemical processes in unstructured data. The following subsections discuss how AI has been applied to study mass transport at the microscale, phase transitions at the mesoscale, and morphological evolution at the macroscale, highlighting both the insights gained and the methodological challenges that remain.

### Mass transport

In battery systems, mass transport involves ion diffusion within electrolytes and electrodes, charge carrier migration across electrode–electrolyte interfaces, and the redistribution of reaction products at these interfaces [43] (Fig. 3b). Several *in situ* techniques have been used to study mass transport, including Raman spectrometry [44], optical imaging [45], neutron imaging [46] and nuclear magnetic resonance (NMR) spectroscopy [47,48]. Among these, scanning TEM with electron energy loss spectroscopy (STEM-EELS) provides a unique advantage by enabling direct nanoscale observation of ion diffusion, such as lithium within intercalation-type electrodes [49]. However, the weak lithium signals in EELS spectra significantly extend the acquisition time required for reliable data, resulting in limited temporal resolution for capturing Li-ion dynamics. Efforts to improve temporal resolution by increasing scanning speeds often exacerbate noise, producing low signal-to-noise ratio maps that fail to clearly represent lithium distributions. To address this trade-off, sparse coding has been proposed as a denoising strategy to improve the interpretability of low-resolution, low-dose EELS data [50]. In this approach, high-dose, high-resolution lithium maps collected under quasi-static conditions are used to extract a dictionary of salient features. This dictionary then guides the reconstruction of faster-acquired but noisier images. While the resulting super-resolved maps qualitatively matched high-fidelity references and revealed spatially anisotropic lithium migration, including lateral movement along electrode interfaces, the generalizability of this method highly depends on the representativeness of the training dictionary. There is concern that important but subtle dynamic patterns not included in the dictionary may be missed during reconstruction. In addition to transport within particles, the formation and evolution of the solid–electrolyte interphase (SEI) and cathode–electrolyte interphase (CEI) are also critical. Recently, the concept of ‘SEI-omics’ has been proposed, involving the construction of a dataset comprising cryogenic transmission electron microscopy (cryo-TEM) images paired with co-localized compositional information [51]. By applying interpretable machine learning models, researchers have decoupled the effects of various SEI components on the structure of lithium-metal single crystals. This study highlights the dominant role of the inner SEI layer, particularly its interfacial properties and ion migration rates, in regulating lithium deposition behavior. However, the nanoscale and buried nature of SEI/CEI makes *in situ* observation challenging, limiting the availability of time-series high-quality data. As a result, AI applications in this area remain scarce, highlighting an important opportunity for future AI-driven insights.

Apart from ion diffusion within electrodes, electrochemical systems often produce complex reaction products, and tracking the redistribution of these products is essential for understanding mass transport mechanisms. For instance, in lithium-metal electrodes, the lithium-related structures include dendrites, pits, deposited lithium and re-deposited lithium. X-ray microscopy provides a non-destructive platform for tracking these structures under realistic operating conditions [52–54]. However, segmentation of lithium phases is challenging due to their low atomic number, which leads to weak X-ray contrast and poor pixel-wise separability. Manually annotating large X-ray datasets is therefore infeasible [55]. To automate this task, CNNs have been adopted for segmenting weakly contrasted X-ray images [56]. One study used a CNN to combine a small number of manual annotations with model-assisted labeling to achieve high accuracy (98.8% on test data). This enabled classification of lithium-related features into five categories and supported downstream analysis of electrode evolution and swelling. While this approach doesn’t need high-quality reference images, model performance still depends on the representativeness of the training data and may deteriorate when encountering previously unseen or rare morphologies. As with many deep learning applications in the physical sciences, incorporating chemical constraints into the model offers promise for improving interpretability.

### Structural transition

Electrochemical driving forces induce phase transitions, defect evolution and coordination changes in electrodes or electrolytes (Fig. 3c) [57]. For example, in high-nickel layered cathodes, lattice oxygen loss during cycling triggers cation mixing, the formation of disordered spinel phases and eventually inactive rock-salt phases [58–60]. Annular dark-field STEM (ADF-STEM) enables atomic-scale visualization of such processes [61]. However, its effectiveness is limited by noise and signal distortions, particularly in regions with high lattice strain, making it difficult to resolve atomic details or delineate phase boundaries under realistic cycling conditions. These challenges necessitate the use of advanced image processing techniques, such as atom segmentation, denoising and structural deblurring.

CNNs, trained on large-scale image datasets, have shown utility in enhancing ADF-STEM image quality by super-resolution processing, atom identification and edge atom localization [62–64]. For example, after training on a TEM image library [65], CNN-based models were used to resolve atomic motifs at the O1–O3 phase boundary in highly delithiated cathodes, revealing shear-induced transformations and lattice distortions [66]. Similarly, CNNs have enabled high-throughput statistical analysis of lattice defect evolution in LiNi_0.5_Co_0.2_Mn_0.3_O_2_ (NCM 523). A dual attention-based CNN identified and classified vacancy and interstitial defects, showing that defect populations initially rise and then decline during degradation. This is a trend correlated with reduced lithium site availability and capacity fade [67]. Despite their promise, their performance is highly dependent on large, well-labeled datasets, which are costly and time-consuming to obtain in materials science. Furthermore, CNNs trained on specific imaging conditions or materials systems may suffer from overfitting and struggle to generalize across different materials or imaging technology settings.

In addition to electrodes, solid electrolytes (SEs) undergo structural evolution during lithiation and delithiation when in contact with the electrode, forming solid–solid interphase layers at the SE–electrode interface [68]. Sulfide-based SEs, compared with oxide-based SEs, have a narrower electrochemical stability window and are prone to decomposition when the potential exceeds this range [69]. Understanding the atomic-scale interface reaction mechanisms is critical for designing more stable SEs and effective buffer layers. X-ray absorption near-edge structure (XANES) spectroscopy provides valuable insights into the oxidation states, site symmetries and covalent bond strengths of specific elements, making it a key tool for studying structural and electronic changes in SEs during cycling [70]. However, interpreting XANES spectra often requires extensive modeling of atomic structures [71]. To aid interpretation, AI has been coupled with density functional theory (DFT)-based spectral simulations to analyze structural evolution in SEs. One strategy involves generating XANES spectra under varying delithiation states, followed by unsupervised clustering to infer correlations between spectral patterns and atomic configurations [72]. This approach enabled the identification of degradation pathways in PS_4_ tetrahedra, including S–S bond formation and distortion into PS_4_-like motifs. While promising, clustering outcomes depend on the chosen distance metrics and dimensionality reductions, potentially biasing structural interpretations.

### Electrode evolution

During electrochemical reactions, electrode particles undergo dynamic changes, including expansion, contraction, morphological reconstruction, aggregation, dispersion and the formation or breakdown of particle interfaces [57]. Over time, these cumulative changes can lead to irreversible damage, especially under high-rate charge/discharge or long-cycle conditions [73]. The mechanisms of electrode evolution are closely tied to the spatial arrangement of electrode components, including the carbon matrix, pores, binder and active particles. To monitor these changes, high spatial resolution and composition-sensitive multiscale visualization techniques, such as X-ray microtomography and nanotomography have been applied [74]. By using the tunable energy of synchrotron X-rays, researchers can reveal the 2D/3D compositional and state-of-charge heterogeneity of electrodes [75,76].

However, conventional X-ray attenuation contrast has limited sensitivity for low atomic number (low Z) elements such as carbon and fluorine, making it difficult to image the carbon/binder domain (CBD) in composite electrodes. Phase-contrast hard X-ray nanotomography improves contrast for low-Z materials by using electron density instead of absorption, and allows the simultaneous imaging of both CBD and high-Z active particles like LiNi_0.8_Mn_0.1_Co_0.1_O_2_ (NMC). In a recent study, this method was combined with a slice-by-slice segmentation model based on CNN to reconstruct thousands of particles in 3D [77]. This approach enabled statistical analysis of particle detachment, showing that particles with smaller sizes or under fast cycling were more likely to lose contact with the conductive matrix. While this workflow helps analyze large volumes of tomography data, several challenges remain. The low-quality phase-retrieved images can still be hard to interpret, especially for complex or damaged regions. The segmentation pipeline requires many manual annotations and assumes that particle shapes change smoothly between slices, which may not hold in all conditions. Looking forward, the strength of transformer-based methods in capturing complex global interactions positions them as promising candidates for more robust 3D segmentation [78].

Beyond individual particle behavior, recent work has examined collective dynamics within particle networks (Fig. 3d). By tracking thousands of NMC particles over time, a shift from disordered to synchronized detachment was observed during extended cycling [79]. To model this behavior, both a regularized encoder and random forest were applied to features including shape, location and local structure. The encoder captured non-linear patterns, while SHAP analysis highlighted that early damage was linked to particle-intrinsic features, whereas later synchronization reflected inter-particle interactions. While informative, these findings rely on feature selection and are limited by model assumptions. The correlation between network behavior and performance remains suggestive, and further mechanistic validation is needed to confirm causality.

To further analyze local crystal structures and microstrains during electrode evolution, micro-focused beam scanning X-ray diffraction computed tomography (μ-XRD CT) has been used. This approach reconstructs phase maps of active particles and the CBD based on unique reflection positions [80]. However, μ-XRD CT generates large datasets, often exceeding 10 000 diffraction patterns per slice, posing challenges for traditionally manual analysis methods. AI addresses this by condensing datasets with PCA and clustering them to identify spatially linked phase distributions (Fig. 3d). Supervised models further refine predictions of spatial phase distributions, using diverse algorithms, including support vector machines, artificial neural networks, k-nearest neighbors and CNNs, to ensure robust and adaptable solutions [81]. This approach has revealed severe structural fatigue on the surfaces of smaller LiNi_0.6_Co_0.2_Mn_0.2_O_2_ particles and larger particles in contact with CBD after prolonged cycling, as well as strain-induced degradation in Li[Li_0.2_Ni_0.2_Mn_0.6_]O_2_ under high-voltage conditions, leading to significant capacity and voltage decay. However, since it is impossible to exhaustively evaluate all plausible models, the reliance on testing multiple models and finding the one with highest accuracy raises concerns about completeness. Moreover, model performance does not necessarily equate to understanding the depth of the chemical mechanism.

Finally, many characterization techniques are destructive, making it difficult to track the same sample over time. To address this, recent work has applied physics-constrained unsupervised image-to-image translation models to infer intermediate structural states from discontinuous data [82].

AI has shown promise in interpreting time-resolved characterization data across multiple scales, offering new perspectives on ion transport, phase transitions and particle-level dynamics. Currently, AI primarily serves as a tool for facilitating acquisition, and statistical analysis of temporal evolution data, supporting rather than replacing human insight. Moving forward, annotating time-sequenced data with specific chemical events and adopting models designed for temporal learning (e.g. recurrent neural networks or transformers) could enable AI to capture not just patterns, but causal sequences within evolution processes. As electrochemical systems are increasingly studied through multimodal and multiscale datasets, a central question emerges: can AI move beyond fitting patterns within isolated domains to uncover relationships across dimensions? The next section will discuss this topic in detail.

## AI FOR CROSS-DIMENSIONAL ELECTROCHEMICAL MECHANISM DISCOVERY

**Figure 4.**
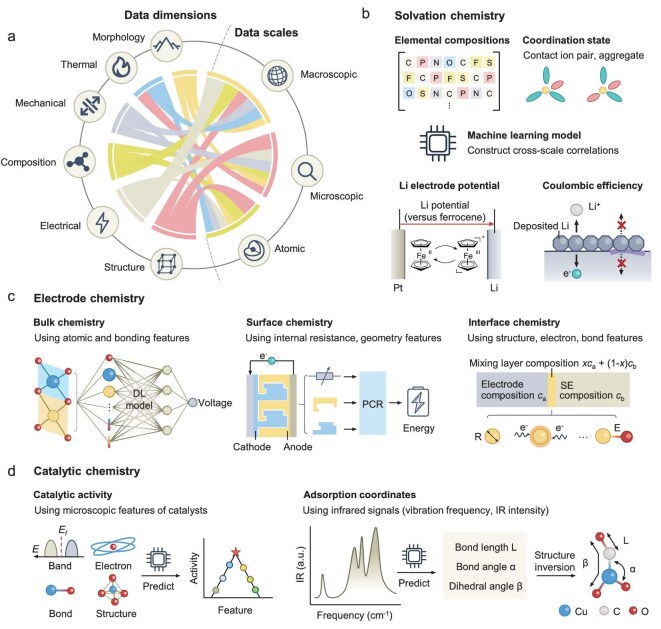
AI for cross-dimensional electrochemical mechanism discovery. (a) Data dimensions and scales in multimodal battery research, spanning atomic to macroscopic levels and incorporating diverse properties. These data types are often interconnected, requiring models that can integrate cross-modal signals. (b) Solvation chemistry: AI models establish cross-scale correlations between micro-level features, such as elemental compositions and coordination states, and macro-level performance metrics, including Li electrode potential and CE. (c) Electrode chemistry: AI constructs relationships between atomic and bonding features and electrode voltage, between internal resistance and geometric features and areal energy density, and between structural, electronic and bonding properties and interface stability. DL, deep learning; PCR, principal component regression. (d) Catalytic chemistry: AI supports catalyst design by linking microscopic features of catalysts to catalytic activity. In addition, machine learning models can extract adsorption coordinates, such as bond lengths and angles, from infrared spectra, to deduce adsorption coordinates and structural configurations.

Multimodal characterization experiments and simulation tools provide a wealth of cross-dimensional data, encompassing composition, structure, morphology, and electrical, mechanical and thermal properties across scales ranging from atomic to macroscopic (Fig. 4a) [83,84]. While such heterogeneity holds the promise of deeper mechanistic insight, traditional analysis methods often treat these modalities separately, overlooking implicit correlations essential for holistic understanding [85]. The core challenge lies in extracting non-linear, high-dimensional and sometimes counterintuitive relationships from inherently heterogeneous datasets. These datasets often differ in spatial and temporal resolution, information content and structure, ranging from spectroscopic signals to imaging features and electrochemical performance. Aligning and integrating such data into a coherent framework exceeds the capacity of most traditional statistical methods.

Recent developments in AI offer potential solutions. Multimodal models can learn joint feature representations across data types and enable the construction of unified frameworks that retain key modality-specific signals while highlighting cross-modal dependencies [86–88]. Interpretable AI methods can help identify which features or combinations drive observed electrochemical behavior, although attention is needed to distinguish physically meaningful patterns from spurious correlations. Such models have begun to reveal relationships not easily discovered by expert intuition. For example, in solvation chemistry, AI has linked elemental compositions and coordination features to Coulombic efficiency (CE) and Li electrode potential. In electrode design, relationships between 3D architecture and performance have been inferred from geometry-resolved descriptors. In catalytic systems, AI-driven structure inversion from infrared spectra has revealed coordination environments of transient species. These cases in the following subsections serve as concrete illustrations of AI’s capacity to move beyond traditional methods toward discovering hidden patterns across electrochemical systems.

### Solvation chemistry

Solvation chemistry studies the solvation structures, (de)solvation dynamics, ion transport in the electrolyte, and interface formation [89]. These factors govern the initial formation and long-term evolution of the electrode–electrolyte interface, which in turn controls key properties such as CE and cycling stability [90]. In lithium-metal batteries, for instance, solvation structures influence electrolyte decomposition pathways and the resulting SEI, which can either passivate or destabilize the Li surface [91].

AI models have been used to examine correlations between electrolyte composition and CE, particularly when traditional approaches fall short in exploring the multiple factors (Fig. 4b). In one example, a dataset encompassing 150 Li|Cu half-cells, comprising 54 solvents and 17 salts across diverse electrolyte formulations, was used to train several AI models including linear regression, random forests and support vector machines [92]. Among these, solvent oxygen content was consistently identified as a dominant feature influencing CE. The proposed rationale is that lower oxygen content may weaken solvation strength, promoting an inorganic-rich SEI favorable for high-efficiency Li plating/stripping. The derived insight is chemically plausible and has guided the design of fluorine-free electrolytes with CE approaching 99.70%, suggesting promising potential under other conditions. But further external validation is still needed. As the current model considers only composition, it cannot distinguish isomers with different atomic arrangements. Future studies should incorporate more molecular details, such as structure, size and functional group type and position, to enhance the robustness and generalizability of AI models.

AI has also contributed to clarifying the mechanistic basis of CE through the theory of Li electrode potential [93]. This quantity, reflecting the reducing ability of Li metal, can be modulated to suppress undesired side reactions. Researchers applied partial least squares regression (PLSR) to a feature set including coordination environment, density, dipole moment, frontier orbital energies and so on. Through this analysis, they identified Li-FSI^−^ coordination as a key factor influencing electrode potential [94]. This result was further corroborated by Raman spectroscopy. Importantly, this analysis demonstrates how interpretable models can help identify dominant variables in a broad space of features.

In addition, researchers have extracted features directly from electrochemical curves to predict failure pathways of lithium-metal anodes. By analyzing the first two cycles of Li|Cu half-cells, they identified correlations between initial lithium deposition/stripping behaviors and subsequent anode degradation. The extracted features relate to the structure of deposited lithium and its interphase with the electrolyte, both of which determine how the anode eventually fails. These structural characteristics influence the formation of interphase regions that lack intimate contact with the lithium metal, as well as the accumulation of inactive lithium. Such defects significantly hinder the transport of charge carriers, including lithium ions and electrons, and play a crucial role in defining the specific mode of failure [95].

To explore the vast molecular design space of potential solvents and achieve a more comprehensive understanding of solvation chemistry, graph-based generative models offer an alternative to empirical trial and error [96]. Here, molecules are represented as graphs composed of atoms and bonds, systematically constructing candidate structures based on scaffold molecules such as formaldehyde and dimethyl ether. One study generated 1399 hypothetical solvents containing up to nine heavy atoms, and applied SHAP to identify dipole moment and molecular radius as key predictors of reductive stability of coordinated solvents. These descriptors align with known chemical trends, bridging molecular structure with coordination stability in ion–solvent complexes.

### Electrode chemistry

Electrode chemistry explores the chemical and electrochemical processes within electrodes during battery operation, focusing on their structure, composition and reactivity [97]. Cross-dimensional analysis of electrode chemistry enables the identification and optimization of key performance factors, including electrode voltage, areal energy density and side reactions (Fig. 4c).

Electrode voltage, defined as the potential difference between an electrode and a reference electrode, reflects the energy output capability of the electrode material under specific conditions. It directly determines the battery’s energy density and its suitability for various applications. The voltage is governed by the intrinsic properties of the electrode material, such as elemental composition and crystal structure. However, the complicated relationships, together with cross-dimensional properties between these factors and the electrode voltage, pose challenges for traditional experimental or calculation methods. AI, particularly deep learning models with a transfer learning strategy, has proved to be effective in addressing these challenges [98]. By applying multilayer neural networks, hierarchical chemical features from crystals were extracted. To predict electrode voltage using elemental and structural features of electrode crystals, Zhang *et al.* transferred the knowledge learned from large-scale datasets (such as Li-ion batteries) to data-scarce systems (such as multivalent-ion batteries) and predicted electrode voltage using elemental and structural features of electrode crystals. They explain how different element groups in the periodic table contribute to voltage by extracting elemental and local environment representations from the embedding and convolutional layers, respectively, and mapping them into two dimensions to visualize atomic contributions to crystal voltage. For instance, the covalent radius of atoms was identified as the most critical feature, providing deeper insights into the fundamental factors influencing electrode chemistry.

Low areal energy density presents a bottleneck for many applications, particularly in microbatteries. While thicker electrodes can increase capacity, they also raise internal resistance due to longer ion-transport distances, hindering improvements in areal energy density. 3D electrode architectures, such as interdigitated-plate configurations, offer a solution by increasing capacity without extending ion-transport distances, thus enhancing areal energy density [99]. However, the relationship between 3D electrode geometries and battery performance remains unclear. Principal component regression (PCR) has been applied to model the influence of structural and electrical descriptors, such as internal resistance, electrode volume and geometry, on energy output [100]. Among these, internal resistance emerged as a dominant factor. While the model’s accuracy supports its utility in design iteration, it operates within a limited configuration space; extrapolation to other architectures or chemistries may require additional constraints.

Side reactions at electrode–electrolyte interfaces, particularly in solid-state batteries, remain a major challenge for long-term stability [8]. AI models have been used to assess the thermodynamic stability of interfaces across a range of electrode and solid electrolyte chemistries [72,101]. For example, automated screening coupled with support vector classification and kernel ridge regression was used to evaluate reaction pathways at the Li|LiLaZrO₇ interface [102]. Among the findings, M–O bond strength was correlated with interfacial reactivity, providing a chemically intuitive link that supports prior empirical observations. While such results underscore the potential of AI models to distill interpretable relationships from high-throughput computations, their effectiveness depends heavily on the quality and coverage of the input data. Moreover, the identification of important features may vary across models, and model-specific interpretations should be treated with caution. Bridging the gap between model outputs and mechanistic understanding remains an open and important challenge.

### Catalytic chemistry

In cross-dimensional chemical mechanism analysis, catalytic chemistry establishes relationships between microscopic features of catalysts (e.g. intrinsic band gaps, outer electron counts) and macroscopic catalytic activity, providing critical insights to understand catalytic origins and facilitate the design of highly active catalysts (Fig. 4d) [103–105].

Recent studies have demonstrated how AI can uncover non-intuitive and universal descriptors from complex, heterogeneous datasets in sulfur reduction reactions (SRRs). In one study, over 2900 historical SRR publications were mined, and features were extracted to train a collaborative learning model that identified a composite descriptor D, dominated by the atomic dispersion factor. This descriptor, which contrasts with conventional electronic-state-based metrics, quantitatively links atom topological arrangements to catalyst–polysulfide interaction strengths. Combined with a volcano relationship between overpotential and interaction strength, this approach enabled the prediction of catalytic activity for over 800 candidates and the discovery of dozens of new SRR catalysts from a pool of 374 833 materials [106]. Complementarily, another study emphasized environment-aware catalyst design, addressing the limitations of fixed catalyst structures under varying electrolyte conditions. Using nickel sulfides as a model system, interpretable machine learning models were applied to relate local chemical environments, which formed by the interplay of solvent and polysulfide species, to interfacial catalytic behavior. The models revealed that optimal catalyst differs depending on whether polysulfide species are dilute or concentrated, guiding the structural modification of Ni_3_S_2_ catalysts for either enhanced ion or electron transport. This adaptive design strategy enabled high-energy pouch cell performance under lean-electrolyte conditions [107].

In addition to theoretical calculations, benefiting from feature engineering, experimentally measurable data, such as spectral data, were identified to offer physically meaningful descriptors [108,109]. For example, in CO_2_ reduction reactions, AI models used infrared signals of the key intermediate CO (vibration frequency, infrared intensity) to predict structural parameters, such as bond lengths, angles and dihedral angles, to locate CO adsorbates on catalysts. These parameters enabled precise determination of CO adsorption coordinates on catalysts, effectively achieving structure inversion [110]. Coupled with ultrafast *in situ* spectroscopy, this workflow enabled dynamic structure inversion and offered a pathway to probe time-resolved catalytic mechanisms based on spectral descriptors. This approach illustrates how AI can infer molecular configurations from macroscopic observables, an inverse problem that is difficult to solve using first-principles methods alone. However, the accuracy of such structural predictions depends heavily on the quality, diversity and resolution of the training data.

In summary, AI enables the identification of meaningful descriptors across high-dimensional chemical parameter spaces, offering an alternative to intuition-driven approaches in cross-dimensional mechanism discovery [21]. This has facilitated progress in areas ranging from solvation structure analysis to catalytic mechanism inference. However, different models are applied to different data types (such as structural, spectral and electrochemical measurements) and later synthesized through human interpretation. While this late-fusion strategy reduces model complexity, it risks overlooking important inter-modal interactions. Alternative methods such as mid- and early-fusion frameworks can be applied to learn joint representations directly from multimodal datasets. Moreover, in some cases, the models are relatively shallow (e.g. linear regressions or tree-based models) potentially limiting their capacity to capture more complex and non-linear relationships. Besides, datasets containing various modalities with known outcomes for training models are still scarce. As research questions grow more complex, efforts have moved beyond identifying cross-dimensional correlations towards disentangling entire mechanism networks. These networks often involve multiple variables that cannot be adequately captured by pairwise relationships alone. The next section examines how AI is being applied to decouple such tightly coupled phenomena.

## AI IN DECOUPLING COMPLEX INTERACTION NETWORKS

Electrochemical behavior in batteries is governed by the tightly coupled interplay of multiple factors, such as thermodynamics, kinetics and mechanical stress [18,111]. Traditional theoretical and experimental approaches often fail to disentangle the whole interaction networks, especially when the effect of one factor depends on the state of others. AI has been proposed as a tool to help decouple such complex systems, identifying which factors matter most and how they work together. Three representative scenarios are discussed in this section: (i) interdependent material components in battery design; (ii) coupled degradation mechanisms in battery aging; and (iii) multivariate effects in catalytic reactions. Each captures a distinct aspect of the broader challenge: how to infer mechanistic networks when data only reflect the final system performance.

### Component dependency analysis

Electrochemical systems, such as electrodes [112–114] and electrolytes [115–117], are composed of multiple interacting components that collectively determine system performance. However, the specific contributions of individual components are often highly coupled, making it challenging to isolate and quantify their individual effects. Adding further complexity, process parameters such as coating thickness and stacking pressure influence these interactions in non-linear and interdependent ways [118–121]. How to quantitatively decouple the independent contributions of each component and parameter of overall system performance remains challenging. Besides this, the vast number of possible combinations of components and process conditions makes traditional experimental methods impractical for exploring the full design space.

**Figure 5.**
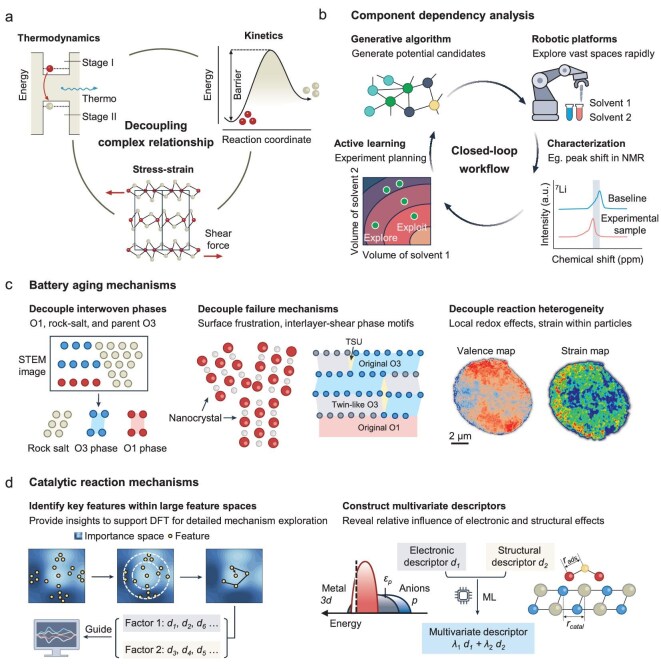
AI-assisted strategies for decoupling complex relationships in battery systems. (a) Electrochemical behaviors are often shaped by interdependent factors such as thermodynamic energy changes, kinetic barriers and stress–strain effects during cycling. These interactions are difficult to separate using traditional analysis. (b) Component dependency analysis: AI-enabled closed-loop workflows integrate generative algorithms for candidate generation, robotic platforms for automated sample preparation and testing, and experimental characterization (e.g. peak shifts in NMR spectra) to evaluate sample performance. These outputs are used by active learning algorithms to guide the selection of the next experimental conditions, enabling efficient exploration of multicomponent design spaces. (c) Battery aging mechanisms: AI techniques help isolate individual degradation modes. This includes separating intergrown phases (e.g. O1, rock-salt, O3), identifying distinct failure origins (e.g. surface frustration vs. interlayer shear) and mapping reaction heterogeneity within particles through valence and strain imaging. TSU: triangle structural unit. (d) Catalytic reaction mechanisms: in high-dimensional catalyst spaces, AI assists in selecting key features that correlate with activity and can guide DFT calculations. It also enables the construction of multivariate descriptors that combine electronic and structural factors to quantify their individual contributions to catalytic performance. Part (c) adapted from ref. [145], American Chemical Society.

AI-based approaches have been increasingly applied to explore vast and intricate parameter spaces (Fig. 5b) [122–124]. By optimizing the composition and proportions of different components, their dependencies can be systematically uncovered. For example, the role of solvents in electrolyte compounds can be revealed during material optimization by generative algorithms [125]. Multiple specific performance criteria, such as chemical stability, conductivity and environmental impact, can be considered via the reinforcement learning and Monte Carlo tree search [112,126]. By combining unsupervised learning and organic electrochemistry, the organic lithium salts with ideal redox activity, decomposition potential, product formation, electrolyte solubility and specific capacity have also been discovered [127]. Deeper mechanistic insights into the optimized materials can be provided by combining these AI methods with advanced characterization techniques and simulations (e.g. DFT) [113].

Integrating active learning techniques, such as Bayesian optimization, with automated high-throughput experimentation platforms in robotic laboratories has accelerated component dependency analysis. These closed-loop workflows allow for rapid exploration of vast design spaces while minimizing the number of experiments required [115,128–134]. By enabling the discovery of uncharted performance regions, this approach makes material optimization and component dependency analysis more efficient and comprehensive [135–137]. Moreover, the high throughput and repeatability of robotic platforms facilitate large-scale hypothesis testing across diverse chemical spaces, advancing our understanding of fundamental mechanisms [138]. When coupled with interpretability-focused techniques, such as feature attribution or symbolic regression, these systems offer the potential to not only optimize, but also understand how compositional variables shape electrochemical behavior [139].

However, limitations remain. Most AI models used in this context prioritize predictive accuracy or search efficiency, often without assessing whether learned dependencies reflect electrochemical mechanisms. Feature importance scores can shift with model choice or data sampling strategy, raising questions about robustness. Also, AI-identified trends may reflect correlations in limited datasets rather than universal causal relationships. For example, models that propose high-performance electrolyte formulations may not distinguish whether observed improvements arise from enhanced solvation structures. A key challenge moving forward is whether AI frameworks for component optimization can evolve beyond empirical search tools to incorporate mechanistic constraints. Addressing this will require shifting from ‘what works’ to ‘why it works,’ integrating domain knowledge and chemical priors into model architectures or active learning objectives. Besides, in closed-loop experimentation and robotic synthesis, human oversight ensures chemical safety and helps refine model objectives. A human-in-the-loop approach may thus offer a safer and more mechanism-informed path than fully autonomous discovery.

### Battery aging mechanisms

Beyond compositional design, battery aging mechanisms involve couplings among structure, stress and chemistry, posing a different kind of decoupling challenge (Fig. 5c). Unlike compositional optimization, where input variables are predefined, battery aging changes with time and is typically observed through sparse and noisy measurements, making it difficult to isolate causal drivers.

Battery failure pathways rarely evolve independently; instead, they tend to interact in coupled and cascading ways. For example, local strain may initiate structural transitions that are then amplified by redox heterogeneity or surface instability [57]. AI-based methods have been applied to assist in unraveling these relationships, particularly in the analysis of high-resolution imaging datasets. For instance, deep-learning-based segmentation has been used to distinguish O1, rock-salt and O3 phases in layered cathodes, quantifying complex phase interactions and revealing the relationships between electrode structural degradation and spatial phase distribution [140]. Further, by combining expert knowledge, AI-assisted super-resolution atomic imaging techniques can disentangle distinct failure mechanisms. For example, in all-solid-state layered cathodes, degradation origins due to surface frustration and interlayer shear-induced phase transformations are decoupled by an AI-aided atom-by-atom phase segmentation technique [141]. Surface mismatch induces surface nanocrystallization and rock-salt transformation, while delithiation-induced interlayer shear produces bulk O1 phases and complex interface/transition patterns, which differ significantly from those observed in liquid electrolyte-based lithium-ion batteries. Additionally, interactions between mechanical deformation and structural phase changes during electrode degradation have been decoupled, showing that mechanical strain localization due to knotted bands promotes O3-to-O1 transitions within the cathode [142].

Similar approaches have been extended to spectroscopic data. Synchrotron X-ray microscopy has proved to be a powerful tool for studying battery aging, revealing mesoscopic redox layering effects in electrode particles, charge state heterogeneity and interactions between surface chemistry and bulk microstructure [143,144]. AI methods are expected to decouple potential mechanisms intertwined among electronic structure, lattice configuration and microstructure during electrode degradation. For instance, AI-based data classification techniques applied to nanoscale-resolution extended X-ray absorption fine structure signals can extract strain distribution by evaluating mismatches in local lattice configurations, thereby decoupling local strain and redox effects within electrode particles and identifying mesoscopic reaction heterogeneity that impacts strain–redox interactions [145].

Despite increasing applications, most models are still trained on static datasets, such as *ex situ* measurements or sparsely sampled data. These fragmented observations make it difficult to trace how degradation processes develop over time. Unsupervised methods can group similar degradation signatures, but it is unclear whether these clusters reflect real electrochemical mechanisms or just statistical variation. Without time-resolved data or experimental labels, such groupings are prone to overinterpretation. Looking ahead, a central challenge is to move from *post hoc* pattern matching to predictive modeling grounded in electrochemical understanding. Can AI identify not just whether a system fails, but what drives that failure and under which conditions? This will require more time-resolved data, models built with embedded electrochemical constraints, and experiments specifically designed to disentangle overlapping degradation mechanisms.

### Catalytic reaction mechanisms

Unlike compositional or degradation-related processes, catalytic reactions are governed by tightly coupled microscopic factors such as electronic structure, surface geometry and coordination environment (Fig. 5d) [146]. Understanding how these descriptors—individually and in combination—influence catalytic performance remains a central challenge in electrocatalysis. Their strong interdependence often obscures causal relationships, making it difficult to disentangle which features are mechanistically relevant and which are merely correlated.

AI has been applied to this challenge by combining pattern recognition with the analytical depth of DFT. For example, in lithium–sulfur batteries, the mechanism of ensemble effects, interactions between multiple active sites in a catalyst, on catalytic activity has been difficult to decipher due to atomic-scale combinatorial complexity and ambiguous activation mechanisms. To address this challenge, an integrated framework combining AI, DFT and electrochemical testing was developed [147]. Multiperspective machine learning models identify key features affecting ensemble effects within the large feature space of multisite catalysts. These features, divided into categories by experts, decouple intersite interaction mechanisms and coordination structure effects, with DFT providing insights into their specific impacts on catalytic activity. Decoupled mechanisms ultimately guide multisite catalyst design, and electrochemical tests validate the theories.

Efforts have also focused on building multivariate descriptors that quantify the contributions of different factors within complex catalytic reaction mechanisms. In lithium–sulfur battery catalysis, for example, machine learning aids in designing binary descriptors to separate and quantify the independent contributions of electronic and structural effects to sulfur reduction kinetics [148]. Specifically, by integrating DFT with Pearson correlation coefficients, band match index and lattice mismatch index are proposed to correlate adsorption energy and reaction barriers, clarifying their individual influence patterns. A linear combination of band match index and lattice mismatch index is then constructed to gain a comprehensive understanding of catalytic mechanisms. Genetic algorithms explore potential combinations of binary descriptors across large parameter spaces, while Monte Carlo simulations ensure statistical confidence in the generated search space and mitigate uncertainties due to random initialization in genetic algorithms. Optimized binary descriptors decouple orbital coupling and lattice distortions affecting catalytic activity, offering theoretical guidance for designing highly active catalysts. Additionally, least absolute shrinkage and selection operator (LASSO) [149] or sure independence screening and sparsifying operator (SISSO) [150] can efficiently yield optimized composite descriptors from extensive mathematical computations and combinations.

AI has enhanced our ability to analyze large computational datasets, but its role in uncovering true catalytic mechanisms remains uncertain. While the accurate interpretation of conclusions and the avoidance of overinterpretation are fundamental to all scientific research, this challenge is particularly critical in AI applications due to the complexity and black-box nature of many advanced models. The interpretation still relies on human intuition, which introduces a subjective element that AI alone cannot resolve. Moving forward, embedding expert knowledge as structured constraints, such as incorporating chemical rules directly into model loss functions, can guide AI toward mechanistically meaningful results. Moreover, adopting structural causal models could help shift analysis from mere correlations to causal inference. Without such integration, AI risks becoming a sophisticated curve-fitting tool rather than a means of scientific discovery.

Box 3. **Task-specific challenges and AI strategies in electrochemistry**

1. Tracking temporal evolution
   Scientific challenges: in data acquisition, the key question is how to capture high-quality, high-time-resolution data to observe the real-time evolution of electrochemical systems. In data analysis, the challenge lies in how to identify chemically meaningful patterns from large time-series datasets, focusing on temporal changes in electrochemical behavior.
   Data characteristics: time-sequenced with causal dependencies, single modality but large in volume and noisy, unstructured and difficult to label.
   Key barriers: the lack of annotated datasets containing ground-truth chemical information, and the challenge of defining proper task labels for chemical processes in unstructured data.
   To improve image quality while maintaining high time resolution, one strategy is to train models to classify low-quality, fast-scan images using a small labeled dataset; another is to use a few high-quality, slow-scan images to guide the reconstruction of clearer fast-scan images.
   To overcome limited labeled chemical data, one strategy is to teach models to replicate expert labeling on routine tasks, enabling automated feature extraction and annotation; another is to use unsupervised methods like clustering or pattern recognition to explore new insights without needing predefined labels.
2. Discovering cross-dimensional mechanism
   Scientific challenges: in electrochemistry, a single scientific question often involves diverse data types. It is difficult to identify meaningful relationships across such varied information relying on fixed assumptions.
   Data characteristics: multimodal and multiscale (varying in form, unit and structure).
   Key barriers: heterogeneous data are hard to compare or combine; different scales and formats make it hard to build a shared analysis framework, and few datasets containing various modalities with known outcomes can be used for training.
   To unify and analyze heterogeneous data, unstructured data can be converted into structured formats that models can understand, for example, using graphs to represent molecular structures; data complexity can be reduced by selecting or designing key features, for example, using PCA or identifying physics-based composite features; and multiple models can be tested to identify the most robust performer for the specific dataset.
3. Decoupling complex interaction networks
   Scientific challenges: many electrochemical processes involve multiple interacting factors. It is difficult to identify which ones matter most, and how they work together.
   Data characteristics: highly coupled variables with complex, often hidden, cause–effect relationships.
   Key barriers: the space of possible variable combinations is large, multiple factors can influence the same outcome, and quantitative cause–effect reasoning is difficult.
   To manage large design spaces with many variable combinations, AI can be combined with automated experiments. For example, use active learning to select the most informative samples and guide robotic experiments efficiently.
   To better understand how multiple factors influence results, one can use image-based or structure-based models to extract detailed atomic or material features, then combine with expert input for qualitative interpretation; alternatively, one can construct composite descriptors from raw data using AI and apply feature importance analysis to find which combinations best explain observed behaviors.

## CONCLUSION AND OUTLOOK

Compared to traditional intuition- or theory-driven methods, AI offers advantages in analyzing high-dimensional, interdependent data and minimizing the limitation of human cognition and domain knowledge. It has demonstrated effectiveness in tackling three challenges in battery chemistry research: tracking temporal evolution; cross-dimensional mechanism discovery; and decoupling complex interaction networks. Box 3 systematically summarizes them in terms of data characteristics, task-specific barriers and corresponding AI-driven strategies.

Despite the existing various AI strategies, collecting multimodal data from the same sample in a single experiment is also crucial. This approach provides comprehensive and continuous data support across multiple characterizations for mechanism analysis. Also, standardized and uniform experimental protocols are significant to ensure data consistency and comparability across studies. For instance, uniform cycling protocols, consistent imaging parameters and harmonized reporting standards can reduce biases in training and evaluation. Besides, open-source data platforms play a critical role in facilitating collaboration and accelerating progress. Shared repositories with well-annotated datasets allow researchers to train generalized AI models that can adapt to diverse battery chemistry research. In addition, careful consideration of ground truth is important. Since AI models are data-driven, their outputs are only as reliable as the reference labels or benchmarks they are trained and evaluated against. Ground truth in battery mechanism research can originate from experimental measurements, computational simulations or expert-curated annotations. Cross-validation across modalities is essential to ensure correctness. Open and community-contributed repositories can further strengthen ground truth by enabling continuous updates and broader coverage. To reflect real-world complexity, these datasets could also include quantified uncertainties rather than single values. Finally, well-defined benchmark tasks built on such datasets will provide fairgrounds for comparing AI methods and advancing mechanism discovery. Furthermore, close collaborations between AI developers and electrochemical domain experts are necessary to protect the relevant chemical insights [151,152]. Expert-guided feature engineering, for example, can emphasize descriptors like coordination state, dipole moment or band match index, vital for interpreting AI predictions, and decide the analysis depth towards the electrochemical mechanism.

Looking forward, the scalability of AI in battery research will benefit from emerging trends in foundational AI models, autonomous agents and advanced reasoning frameworks. Large language models (LLMs) [153,154], inspired by advancements in natural language processing, could serve as universal tools for battery research, providing generalized insights across diverse datasets and applications [155]. These models would reduce the reliance on task-specific approaches, enabling more efficient research workflows. However, their current applications in the battery field have largely focused on literature mining [156], data extraction [157,158] and state of charge (SOC) estimation [159], rather than on uncovering underlying electrochemical mechanisms. They face several key limitations, including interpretability challenges, high data demands and a mismatch between battery data modalities (e.g. molecular structure, spectra and electrochemical performance) and the text-based nature of LLMs. Despite these challenges, recent progress, such as domain-adapted pretraining, reinforcement learning-based reasoning, multimodal fusion and knowledge distillation, suggests a promising path forward. Addressing data scarcity through public dataset-sharing and benchmark development will also be critical for utilizing the full potential of LLMs in this field. Moreover, autonomous AI agents integrated with robotic experimentation platforms hold the potential to revolutionize materials optimization and mechanism discovery [160]. By autonomously designing, conducting and analyzing experiments in closed-loop workflows, these systems distill generalizable chemical principles and accelerate discovery cycles while reducing experimental costs. However, the deployment of such systems must be guided by carefully designed human–AI collaboration frameworks that preserve human oversight in hypothesis generation, risk assessment and ethical judgment, where current AI systems still lack contextual understanding. Finally, incorporating causal inference [161] and physics-informed principles into AI models will enhance their interpretability and ensure alignment with established electrochemical theories, bridging the gap between correlation and causation [162–164].

## References

[1] Zuo W, Gim J, Li T et al. Microstrain screening towards defect-less layered transition metal oxide cathodes. Nat Nanotechnol 2024; 19: 1644–53. 10.1038/s41565-024-01734-x 39164411

[2] Xu Q, Li T, Ju Z et al. Li_2_ZrF_6_-based electrolytes for durable lithium metal batteries. Nature 2025; 637: 339–46. 10.1038/s41586-024-08294-z 39780011

[3] Lee J, Zhou S, Ferrari VC et al. Halide segregation to boost all-solid-state lithium-chalcogen batteries. Science 2025; 388: 724–9. 10.1126/science.adt1882 40373146

[4] Huang Y, Dong Y, Yang Y et al. Integrated rocksalt–polyanion cathodes with excess lithium and stabilized cycling. Nat Energy 2024; 9: 1497–505. 10.1038/s41560-024-01615-6

[5] Choi G, Sullivan P, Lv X-L et al. Soft–hard zwitterionic additives for aqueous halide flow batteries. Nature 2024; 635: 89–95. 10.1038/s41586-024-08079-4 39443802

[6] Yi X, Fu H, Rao AM et al. Safe electrolyte for long-cycling alkali-ion batteries. Nat Sustain 2024; 7: 326–37. 10.1038/s41893-024-01275-0

[7] Innocenti A, Beringer S, Passerini S. Cost and performance analysis as a valuable tool for battery material research. Nat Rev Mater 2024; 9: 347–57. 10.1038/s41578-024-00657-2

[8] Wan H, Xu J, Wang C. Designing electrolytes and interphases for high-energy lithium batteries. Nat Rev Chem 2024; 8: 30–44. 10.1038/s41570-023-00557-z 38097662

[9] Wang Z, Xia J, Ji X et al. Lithium anode interlayer design for all-solid-state lithium-metal batteries. Nat Energy 2024; 9: 251–62. 10.1038/s41560-023-01426-1

[10] Wen S, Sun Z, Wu X et al. Regulating interfacial chemistry to boost ionic transport and interface stability of composite solid-state electrolytes for high-performance solid-state lithium metal batteries. Adv Funct Mater 2025; 35: 2422147. 10.1002/adfm.202422147

[11] Lu D, Li R, Rahman MM et al. Ligand-channel-enabled ultrafast Li-ion conduction. Nature 2024; 627: 101–7. 10.1038/s41586-024-07045-4 38418886

[12] Liu R, Wei Z, Peng L et al. Establishing reaction networks in the 16-electron sulfur reduction reaction. Nature 2024; 626: 98–104. 10.1038/s41586-023-06918-4 38297176

[13] Sun Z, Zhang Z, Zhou S et al. Effective binding sufficiently-small SiO_2_ nanoparticles within carbon nanosheets framework enables a high-performing and durable anode for lithium-ion batteries. J Materiomics 2025; 11: 101053. 10.1016/j.jmat.2025.101053

[14] Zhou S, Sun Z, Zhuang Z et al. Facile and scalable synthesis of bismuth oxyhalide nanosheets anodes for fast and durable sodium-ion storage. Sci China Mater 2025; 68: 868–78. 10.1007/s40843-024-3175-3

[15] Zhang Y, Hu A, Xia D et al. Operando characterization and regulation of metal dissolution and redeposition dynamics near battery electrode surface. Nat Nanotechnol 2023; 18: 790–7. 10.1038/s41565-023-01367-6 37081082

[16] Gervillié-Mouravieff C, Bao W, Steingart DA et al. Non-destructive characterization techniques for battery performance and life-cycle assessment. Nat Rev Electr Eng 2024; 1: 547–58. 10.1038/s44287-024-00069-y

[17] Ning Z, Li G, Melvin DLR et al. Dendrite initiation and propagation in lithium metal solid-state batteries. Nature 2023; 618: 287–93. 10.1038/s41586-023-05970-4 37286650

[18] Zhao H, Deng HD, Cohen AE et al. Learning heterogeneous reaction kinetics from X-ray videos pixel by pixel. Nature 2023; 621: 289–94. 10.1038/s41586-023-06393-x 37704764 PMC10499602

[19] Wang H, Fu T, Du Y et al. Scientific discovery in the age of artificial intelligence. Nature 2023; 620: 47–60. 10.1038/s41586-023-06221-2 37532811

[20] Lombardo T, Duquesnoy M, El-Bouysidy H et al. Artificial intelligence applied to battery research: hype or reality? Chem Rev 2022; 122: 10899–969. 10.1021/acs.chemrev.1c00108 34529918 PMC9227745

[21] Peng J, Schwalbe-Koda D, Akkiraju K et al. Human- and machine-centred designs of molecules and materials for sustainability and decarbonization. Nat Rev Mater 2022; 7: 991–1009. 10.1038/s41578-022-00466-5

[22] Yao ZP, Lum YW, Johnston A et al. Machine learning for a sustainable energy future. Nat Rev Mater 2023; 8: 202–15. 10.1038/s41578-022-00490-5 36277083 PMC9579620

[23] Li J, Zhou M, Wu H-H et al. Machine learning-assisted property prediction of solid-state electrolyte. Adv Energy Mater 2024; 14: 2304480. 10.1002/aenm.202304480

[24] Mou T, Pillai HS, Wang S et al. Bridging the complexity gap in computational heterogeneous catalysis with machine learning. Nat Catal 2023; 6: 122–36. 10.1038/s41929-023-00911-w

[25] Esterhuizen JA, Goldsmith BR, Linic S. Interpretable machine learning for knowledge generation in heterogeneous catalysis. Nat Catal 2022; 5: 175–84. 10.1038/s41929-022-00744-z

[26] Zheng ZY, Zhou J, Zhu YS. Computational approach inspired advancements of solid-state electrolytes for lithium secondary batteries: from first-principles to machine learning. Chem Soc Rev 2024; 53: 3134–66. 10.1039/D3CS00572K 38375570

[27] Hippalgaonkar K, Li Q, Wang X et al. Knowledge-integrated machine learning for materials: lessons from gameplaying and robotics. Nat Rev Mater 2023; 8: 241–60. 10.1038/s41578-022-00513-1

[28] Tao H, Wu T, Aldeghi M et al. Nanoparticle synthesis assisted by machine learning. Nat Rev Mater 2021; 6: 701–16. 10.1038/s41578-021-00337-5

[29] Suvarna M, Pérez-Ramírez J. Embracing data science in catalysis research. Nat Catal 2024; 7: 624–35. 10.1038/s41929-024-01150-3

[30] Thon C, Finke B, Kwade A et al. Artificial intelligence in process engineering. Adv Intell Syst 2021; 3: 2000261. 10.1002/aisy.202000261

[31] Hu E, Choo HH, Zhang W et al. Integrating machine learning and characterization in battery research: toward cognitive digital twins with physics and knowledge. Adv Funct Mater 2025; 35: 2422601. 10.1002/adfm.202422601

[32] Hu X, Xu L, Lin X et al. Battery lifetime prognostics. Joule 2020; 4: 310–46. 10.1016/j.joule.2019.11.018

[33] Tao S, Liu H, Sun C et al. Collaborative and privacy-preserving retired battery sorting for profitable direct recycling via federated machine learning. Nat Commun 2023; 14: 8032. 10.1038/s41467-023-43883-y 38052823 PMC10697957

[34] Tao S, Ma R, Zhao Z et al. Generative learning assisted state-of-health estimation for sustainable battery recycling with random retirement conditions. Nat Commun 2024; 15: 10154. 10.1038/s41467-024-54454-0 39578484 PMC11584641

[35] Gilpin LH, Bau D, Yuan BZ et al. Explaining explanations: an overview of interpretability of machine learning. In: 2018 IEEE 5th International Conference on Data Science and Advanced Analytics (DSAA), Turin, Italy, 1–3 October 2018. pp. 80–9. IEEE. 10.1109/DSAA.2018.00018

[36] Merryweather AJ, Jacquet Q, Emge SP et al. Operando monitoring of single-particle kinetic state-of-charge heterogeneities and cracking in high-rate Li-ion anodes. Nat Mater 2022; 21: 1306–13. 10.1038/s41563-022-01324-z 35970962

[37] Miele E, Dose WM, Manyakin I et al. Hollow-core optical fibre sensors for operando Raman spectroscopy investigation of Li-ion battery liquid electrolytes. Nat Commun 2022; 13: 1651. 10.1038/s41467-022-29330-4 35347137 PMC8960792

[38] Chen Y, Wang Z, Li X et al. Li metal deposition and stripping in a solid-state battery via Coble creep. Nature 2020; 578: 251–5. 10.1038/s41586-020-1972-y 32015545

[39] Xu Y, Jia H, Gao P et al. Direct in situ measurements of electrical properties of solid–electrolyte interphase on lithium metal anodes. Nat Energy 2023; 8: 1345–54. 10.1038/s41560-023-01361-1 38249622 PMC10798234

[40] Zhou S, Shi J, Liu S et al. Visualizing interfacial collective reaction behaviour of Li–S batteries. Nature 2023; 621: 75–81. 10.1038/s41586-023-06326-8 37673990

[41] Zhang D, Wang R, Wang X et al. In situ monitoring redox processes in energy storage using UV–Vis spectroscopy. Nat Energy 2023; 8: 567–76. 10.1038/s41560-023-01240-9

[42] Zhang Q, Song Z, Sun X et al. Atomic dynamics of electrified solid–liquid interfaces in liquid-cell TEM. Nature 2024; 630: 643–7. 10.1038/s41586-024-07479-w 38898295

[43] Jun K, Chen Y, Wei G et al. Diffusion mechanisms of fast lithium-ion conductors. Nat Rev Mater 2024; 9: 887–905. 10.1038/s41578-024-00715-9

[44] Liu J, Hua H, Lin J et al. Optimizing interface concentration and electric fields for enhanced lithium deposition behavior in lithium metal anodes. Energy Environ Sci 2024; 17: 5993–6002. 10.1039/D4EE01816H

[45] Zeng C, Chen J, Yang H et al. Visualizing fast interlayer anisotropic lithium diffusion via single crystal microbattery. Matter 2022; 5: 4015–28. 10.1016/j.matt.2022.08.003

[46] Wang C, Wang R, Huang Z et al. Unveiling the migration behavior of lithium ions in NCM/Graphite full cell via *in operando* neutron diffraction. Energy Stor Mater 2022; 44: 1–9. 10.1016/j.ensm.2021.09.032

[47] Liang Z, Xiang Y, Wang K et al. Understanding the failure process of sulfide-based all-solid-state lithium batteries via operando nuclear magnetic resonance spectroscopy. Nat Commun 2023; 14: 259. 10.1038/s41467-023-35920-7 36650152 PMC9845218

[48] Xiang Y, Tao M, Zhong G et al. Quantitatively analyzing the failure processes of rechargeable Li metal batteries. Sci Adv 2021; 7: eabj3423. 10.1126/sciadv.abj3423 34757793 PMC8580315

[49] Nomura Y, Yamamoto K, Hirayama T et al. Quantitative operando visualization of electrochemical reactions and Li ions in all-solid-state batteries by STEM-EELS with hyperspectral image analyses. Nano Lett 2018; 18: 5892–8. 10.1021/acs.nanolett.8b02587 30130410

[50] Nomura Y, Yamamoto K, Fujii M et al. Dynamic imaging of lithium in solid-state batteries by operando electron energy-loss spectroscopy with sparse coding. Nat Commun 2020; 11: 2824. 10.1038/s41467-020-16622-w 32499493 PMC7272654

[51] Lu G, Han Z, Shi L et al. Decoding single-crystal lithium growth through solid electrolyte interphase omics. Nat Commun 2025; 16: 9323. 10.1038/s41467-025-62166-2 41125574 PMC12546825

[52] Sadd M, Xiong S, Bowen JR et al. Investigating microstructure evolution of lithium metal during plating and stripping via operando X-ray tomographic microscopy. Nat Commun 2023; 14: 854. 10.1038/s41467-023-36568-z 36792892 PMC9931753

[53] Pan H, Fu T, Zan G et al. Fast Li plating behavior probed by X-ray computed tomography. Nano Lett 2021; 21: 5254–61. 10.1021/acs.nanolett.1c01389 34105964

[54] Lewis JA, Cortes FJQ, Liu Y et al. Linking void and interphase evolution to electrochemistry in solid-state batteries using operando X-ray tomography. Nat Mater 2021; 20: 503–10. 10.1038/s41563-020-00903-2 33510445

[55] Scharf J, Chouchane M, Finegan DP et al. Bridging nano- and microscale X-ray tomography for battery research by leveraging artificial intelligence. Nat Nanotechnol 2022; 17: 446–59. 10.1038/s41565-022-01081-9 35414116

[56] Huang Y, Perlmutter D, Su AFH et al. Detecting lithium plating dynamics in a solid-state battery with operando X-ray computed tomography using machine learning. npj Comput Mater 2023; 9: 93. 10.1038/s41524-023-01039-y

[57] Dong Y, Li J. Oxide cathodes: functions, instabilities, self healing, and degradation mitigations. Chem Rev 2023; 123: 811–33. 10.1021/acs.chemrev.2c00251 36398933

[58] Xu C, Märker K, Lee J et al. Bulk fatigue induced by surface reconstruction in layered Ni-rich cathodes for Li-ion batteries. Nat Mater 2021; 20: 84–92. 10.1038/s41563-020-0767-8 32839589

[59] Wang C, Han L, Zhang R et al. Resolving atomic-scale phase transformation and oxygen loss mechanism in ultrahigh-nickel layered cathodes for cobalt-free lithium-ion batteries. Matter 2021; 4: 2013–26. 10.1016/j.matt.2021.03.012

[60] Hu E, Yu X, Lin R et al. Evolution of redox couples in Li- and Mn-rich cathode materials and mitigation of voltage fade by reducing oxygen release. Nat Energy 2018; 3: 690–8. 10.1038/s41560-018-0207-z

[61] Ding F, Ji P, Han Z et al. Tailoring planar strain for robust structural stability in high-entropy layered sodium oxide cathode materials. Nat Energy 2024; 9: 1529–39. 10.1038/s41560-024-01616-5

[62] Esteva A, Chou K, Yeung S et al. Deep learning-enabled medical computer vision. npj Digit Med 2021; 4: 5. 10.1038/s41746-020-00376-2 33420381 PMC7794558

[63] Choudhary K, DeCost B, Chen C et al. Recent advances and applications of deep learning methods in materials science. npj Comput Mater 2022; 8: 59. 10.1038/s41524-022-00734-6

[64] Yang S-H, Choi W, Cho BW et al. Deep learning-assisted quantification of atomic dopants and defects in 2D materials. Adv Sci 2021; 8: 2101099. 10.1002/advs.202101099 PMC8373156 34081415

[65] Lin R, Zhang R, Wang C et al. TEMImageNet training library and AtomSegNet deep-learning models for high-precision atom segmentation, localization, denoising, and deblurring of atomic-resolution images. Sci Rep 2021; 11: 5386. 10.1038/s41598-021-84499-w 33686158 PMC7940611

[66] Wang C, Wang X, Zhang R et al. Resolving complex intralayer transition motifs in high-Ni-content layered cathode materials for lithium-ion batteries. Nat Mater 2023; 22: 235–41. 10.1038/s41563-022-01461-5 36702885

[67] Sha WX, Guo YQ, Cheng DP et al. Degradation mechanism analysis of LiNi_0.5_Co_0.2_Mn_0.3_O_2_ single crystal cathode materials through machine learning. npj Comput Mater 2022; 8: 223. 10.1038/s41524-022-00905-5

[68] He B, Zhang F, Xin Y et al. Halogen chemistry of solid electrolytes in all-solid-state batteries. Nat Rev Chem 2023; 7: 826–42. 37833403 10.1038/s41570-023-00541-7

[69] Janek J, Zeier WG. Challenges in speeding up solid-state battery development. Nat Energy 2023; 8: 230–40. 10.1038/s41560-023-01208-9

[70] Xiang Y, Li X, Cheng Y et al. Advanced characterization techniques for solid state lithium battery research. Mater Today 2020; 36: 139–57. 10.1016/j.mattod.2020.01.018

[71] Qian G, Wang J, Li H et al. Structural and chemical evolution in layered oxide cathodes of lithium-ion batteries revealed by synchrotron techniques. Natl Sci Rev 2022; 9: nwab146. 10.1093/nsr/nwab146 35145703 PMC8824737

[72] Cao CT, Carbone MR, Komurcuoglu C et al. Atomic insights into the oxidative degradation mechanisms of sulfide solid electrolytes. Cell Rep Phys Sci 2024; 5: 101909. 10.1016/j.xcrp.2024.101909

[73] Liu T, Liu J, Li L et al. Origin of structural degradation in Li-rich layered oxide cathode. Nature 2022; 606: 305–12. 10.1038/s41586-022-04689-y 35676429

[74] Xue ZC, Li JZ, Pianetta P et al. Data-driven lithium-ion battery cathode research with state-of- the-art synchrotron X-ray techniques. Acc Mater Res 2022; 3: 854–65. 10.1021/accountsmr.2c00098

[75] Lin F, Nordlund D, Li Y et al. Metal segregation in hierarchically structured cathode materials for high-energy lithium batteries. Nat Energy 2016; 1: 15004. 10.1038/nenergy.2015.4

[76] Lim J, Li Y, Alsem DH et al. Origin and hysteresis of lithium compositional spatiodynamics within battery primary particles. Science 2016; 353: 566–71. 10.1126/science.aaf4914 27493180

[77] Jiang ZS, Li JZ, Yang Y et al. Machine-learning-revealed statistics of the particle-carbon/binder detachment in lithium-ion battery cathodes. Nat Commun 2020; 11: 2310. 10.1038/s41467-020-16233-5 32385347 PMC7210251

[78] Li X, Ding H, Yuan H et al. Transformer-based visual segmentation: a survey. IEEE Trans Pattern Anal Mach Intell 2024; 46: 10138–63. 10.1109/TPAMI.2024.3434373 39074008

[79] Li J, Sharma N, Jiang Z et al. Dynamics of particle network in composite battery cathodes. Science 2022; 376: 517–21. 10.1126/science.abm8962 35482882

[80] Finegan DP, Vamvakeros A, Tan C et al. Spatial quantification of dynamic inter and intra particle crystallographic heterogeneities within lithium ion electrodes. Nat Commun 2020; 11: 631. 10.1038/s41467-020-14467-x 32005812 PMC6994469

[81] Hua W, Chen J, Ferreira Sanchez D et al. Probing particle-carbon/binder degradation behavior in fatigued layered cathode materials through machine learning aided diffraction tomography. Angew Chem Int Ed 2024; 63: e202403189. 10.1002/anie.202403189 38701048

[82] Sciazko A, Komatsu Y, Shimura T et al. Prediction of electrode microstructure evolutions with physically constrained unsupervised image-to-image translation networks. npj Comput Mater 2024; 10: 49. 10.1038/s41524-024-01228-3

[83] Lu J, Wu T, Amine K. State-of-the-art characterization techniques for advanced lithium-ion batteries. Nat Energy 2017; 2: 17011. 10.1038/nenergy.2017.11

[84] Huang J, Boles ST, Tarascon J-M. Sensing as the key to battery lifetime and sustainability. Nat Sustain 2022; 5: 194–204. 10.1038/s41893-022-00859-y

[85] Steyaert S, Pizurica M, Nagaraj D et al. Multimodal data fusion for cancer biomarker discovery with deep learning. Nat Mach Intell 2023; 5: 351–62. 10.1038/s42256-023-00633-5 37693852 PMC10484010

[86] Sheriff K, Cao Y, Smidt T et al. Quantifying chemical short-range order in metallic alloys. Proc Natl Acad Sci USA 2024; 121: e2322962121. 10.1073/pnas.2322962121 38870054 PMC11194554

[87] Ma YR, Jin TW, Choudhury R et al. Understanding the correlation between lithium dendrite growth and local material properties by machine learning. J Electrochem Soc 2021; 168: 090523. 10.1149/1945-7111/ac201d

[88] Zhang Y, Lin XY, Zhai WB et al. Machine learning on microstructure-property relationship of lithium-ion conducting oxide solid electrolytes. Nano Lett 2024; 24: 5292–300. 10.1021/acs.nanolett.4c00902 38648075

[89] Xiao P, Yun X, Chen Y et al. Insights into the solvation chemistry in liquid electrolytes for lithium-based rechargeable batteries. Chem Soc Rev 2023; 52: 5255–316. 10.1039/D3CS00151B 37462967

[90] He X, Bresser D, Passerini S et al. The passivity of lithium electrodes in liquid electrolytes for secondary batteries. Nat Rev Mater 2021; 6: 1036–52. 10.1038/s41578-021-00345-5

[91] Yu W, Lin K-Y, Boyle DT et al. Electrochemical formation of bis(fluorosulfonyl)imide-derived solid-electrolyte interphase at Li-metal potential. Nat Chem 2024; 17: 246–55. 10.1038/s41557-024-01689-5 39622915

[92] Kim SC, Oyakhire ST, Athanitis C et al. Data-driven electrolyte design for lithium metal anodes. Proc Natl Acad Sci USA 2023; 120: e2214357120. 10.1073/pnas.2214357120 36848560 PMC10013853

[93] Kim SC, Kong X, Vilá RA et al. Potentiometric measurement to probe solvation energy and its correlation to lithium battery cyclability. J Am Chem Soc 2021; 143: 10301–8. 10.1021/jacs.1c03868 34184873

[94] Ko S, Obukata T, Shimada T et al. Electrode potential influences the reversibility of lithium-metal anodes. Nat Energy 2022; 7: 1217–24. 10.1038/s41560-022-01144-0

[95] Piao Z, Han Z, Tao S et al. Deciphering failure paths in lithium metal anodes by electrochemical curve fingerprints. Natl Sci Rev 2025; 12: nwaf158. 10.1093/nsr/nwaf158 40511368 PMC12153722

[96] Chen X, Shen X, Hou T-Z et al. Ion-solvent chemistry-inspired cation-additive strategy to stabilize electrolytes for sodium-metal batteries. Chem 2020; 6: 2242–56. 10.1016/j.chempr.2020.06.036

[97] Jain R, Lakhnot AS, Bhimani K et al. Nanostructuring versus microstructuring in battery electrodes. Nat Rev Mater 2022; 7: 736–46. 10.1038/s41578-022-00454-9

[98] Zhang XY, Zhou J, Lu J et al. Interpretable learning of voltage for electrode design of multivalent metal-ion batteries. npj Comput Mater 2022; 8: 175. 10.1038/s41524-022-00858-9

[99] Long JW, Dunn B, Rolison DR et al. 3D architectures for batteries and electrodes. Adv Energy Mater 2020; 10: 2002457. 10.1002/aenm.202002457

[100] Miyamoto K, Broderick S, Rajan K. Three-dimensional microbattery design via an automatic geometry generator and machine-learning-based performance simulator. Cell Rep Phys Sci 2021; 2: 100504. 10.1016/j.xcrp.2021.100504

[101] Ren FC, Wu YQ, Zuo WH et al. Visualizing the SEI formation between lithium metal and solid-state electrolyte. Energy Environ Sci 2024; 17: 2743–52. 10.1039/D3EE03536K

[102] Liu B, Yang J, Yang HL et al. Rationalizing the interphase stability of Li|doped-Li_7_La_3_Zr_2_O_12_ via automated reaction screening and machine learning. J Mater Chem A 2019; 7: 19961–9. 10.1039/C9TA06748E

[103] Han Z, Zhao S, Xiao J et al. Engineering *d*-*p* orbital hybridization in single-atom metal-embedded three-dimensional electrodes for Li–S batteries. Adv Mater 2021; 33: e2105947. 10.1002/adma.202105947 34569660

[104] Lao Z, Han Z, Ma J et al. Band structure engineering and orbital orientation control constructing dual active sites for efficient sulfur redox reaction. Adv Mater 2023; 36: e2309024. 10.1002/adma.202309024 37848387

[105] Han Z, Gao R, Jia Y et al. Catalytic effect in Li-S batteries: from band theory to practical application. Mater Today 2022; 57: 84–120. 10.1016/j.mattod.2022.05.017

[106] Han Z, Tao S, Jia Y et al. Data-driven insight into the universal structure–property relationship of catalysts in lithium–sulfur batteries. J Am Chem Soc 2025; 147: 22851–63. 10.1021/jacs.5c04960 40549489

[107] Jia Y, Wang Z, Han Z et al. Variable and intelligent catalyst design based on local chemical environments in sulfur redox reactions. Joule 2025; 9: 101878. 10.1016/j.joule.2025.101878

[108] Hua W, Shang T, Li H et al. Optimizing the *p* charge of S in *p*-block metal sulfides for sulfur reduction electrocatalysis. Nat Catal 2023; 6: 174–84. 10.1038/s41929-023-00912-9

[109] Huang J, Sementa L, Liu Z et al. Experimental Sabatier plot for predictive design of active and stable Pt-alloy oxygen reduction reaction catalysts. Nat Catal 2022; 5: 513–23. 10.1038/s41929-022-00797-0

[110] Yang T, Zhou D, Ye S et al. Catalytic structure design by AI generating with spectroscopic descriptors. J Am Chem Soc 2023; 145: 26817–23. 10.1021/jacs.3c09299 38019281

[111] Yuan X, Liu B, Mecklenburg M et al. Ultrafast deposition of faceted lithium polyhedra by outpacing SEI formation. Nature 2023; 620: 86–91. 10.1038/s41586-023-06235-w 37532813

[112] Sowndarya SVS, Law JN, Tripp CE et al. Multi-objective goal-directed optimization of de novo stable organic radicals for aqueous redox flow batteries. Nat Mach Intell 2022; 4: 720–30. 10.1038/s42256-022-00506-3

[113] Ogihara N, Hasegawa M, Kumagai H et al. Heterogeneous intercalated metal-organic framework active materials for fast-charging non-aqueous Li-ion capacitors. Nat Commun 2023; 14: 1472. 10.1038/s41467-023-37120-9 36928582 PMC10020440

[114] Wang YD, Meyer Q, Tang K et al. Large-scale physically accurate modelling of real proton exchange membrane fuel cell with deep learning. Nat Commun 2023; 14: 745. 10.1038/s41467-023-35973-8 36788206 PMC9929041

[115] Dave A, Mitchell J, Burke S et al. Autonomous optimization of non-aqueous Li-ion battery electrolytes via robotic experimentation and machine learning coupling. Nat Commun 2022; 13: 5454. 10.1038/s41467-022-32938-1 36167832 PMC9515088

[116] Schmidt J, Wang HC, Schmidt G et al. Machine learning guided high-throughput search of non-oxide garnets. npj Comput Mater 2023; 9: 63. 10.1038/s41524-023-01009-4

[117] Lu G, Qiao Q, Zhang M et al. High-voltage electrosynthesis of organic-inorganic hybrid with ultrahigh fluorine content toward fast Li-ion transport. Sci Adv 2024; 10: eado7348. 10.1126/sciadv.ado7348 39110803 PMC11305396

[118] Zhang X, Luo CQ, Menga N et al. Pressure and polymer selections for solid-state batteries investigated with high-throughput simulations. Cell Rep Phys Sci 2023; 4: 101328.

[119] Kench S, Squires I, Dahari A et al. Li-ion battery design through microstructural optimization using generative AI. Matter 2024; 7: 4260–9. 10.1016/j.matt.2024.08.014

[120] Chen YT, Duquesnoy M, Tan DHS et al. Fabrication of high-quality thin solid-state electrolyte films assisted by machine learning. ACS Energy Lett 2021; 6: 1639–48. 10.1021/acsenergylett.1c00332

[121] Lu G, Nai J, Luan D et al. Surface engineering toward stable lithium metal anodes. Sci Adv 2023; 9: eadf1550. 10.1126/sciadv.adf1550 37018409 PMC10075991

[122] Merchant A, Batzner S, Schoenholz SS et al. Scaling deep learning for materials discovery. Nature 2023; 624: 80–5. 10.1038/s41586-023-06735-9 38030720 PMC10700131

[123] Xie T, France-Lanord A, Wang YM et al. Accelerating amorphous polymer electrolyte screening by learning to reduce errors in molecular dynamics simulated properties. Nat Commun 2022; 13: 3415. 10.1038/s41467-022-30994-1 35701416 PMC9197847

[124] Li K, Wang JF, Song YY et al. Machine learning-guided discovery of ionic polymer electrolytes for lithium metal batteries. Nat Commun 2023; 14: 2789. 10.1038/s41467-023-38493-7 37188717 PMC10185508

[125] Coley CW . Defining and exploring chemical spaces. Trends Chem 2021; 3: 133–45. 10.1016/j.trechm.2020.11.004

[126] Jensen JH . A graph-based genetic algorithm and generative model/Monte Carlo tree search for the exploration of chemical space. Chem Sci 2019; 10: 3567–72. 10.1039/C8SC05372C 30996948 PMC6438151

[127] Chen S, Wu G, Jiang H et al. External Li supply reshapes Li deficiency and lifetime limit of batteries. Nature 2025; 638: 676–83. 10.1038/s41586-024-08465-y 39939772

[128] Noh J, Doan HA, Job H et al. An integrated high-throughput robotic platform and active learning approach for accelerated discovery of optimal electrolyte formulations. Nat Commun 2024; 15: 2757. 10.1038/s41467-024-47070-5 38553488 PMC10980761

[129] Liang YG, Job H, Feng RZ et al. High-throughput solubility determination for data-driven materials design and discovery in redox flow battery research. Cell Rep Phys Sci 2023; 4: 101633. 10.1016/j.xcrp.2023.101633

[130] Eng AYS, Soni CB, Lum YW et al. Theory-guided experimental design in battery materials research. Sci Adv 2022; 8: eabm2422. 10.1126/sciadv.abm2422 35544561 PMC9094674

[131] Matsuda S, Lambard G, Sodeyama K. Data-driven automated robotic experiments accelerate discovery of multi-component electrolyte for rechargeable Li–O_2_ batteries. Cell Rep Phys Sci 2022; 3: 100832. 10.1016/j.xcrp.2022.100832

[132] Dave A, Mitchell J, Kandasamy K et al. Autonomous discovery of battery electrolytes with robotic experimentation and machine learning. Cell Rep Phys Sci 2022; 1: 100264. 10.1016/j.xcrp.2020.100264

[133] Zhong M, Tran K, Min Y et al. Accelerated discovery of CO_2_ electrocatalysts using active machine learning. Nature 2020; 581: 178–83. 10.1038/s41586-020-2242-8 32405017

[134] Szymanski NJ, Rendy B, Fei Y et al. An autonomous laboratory for the accelerated synthesis of novel materials. Nature 2023; 624: 86–91. 10.1038/s41586-023-06734-w 38030721 PMC10700133

[135] Boiko DA, MacKnight R, Kline B et al. Autonomous chemical research with large language models. Nature 2023; 624: 570–8. 10.1038/s41586-023-06792-0 38123806 PMC10733136

[136] Slattery A, Wen Z, Tenblad P et al. Automated self-optimization, intensification, and scale-up of photocatalysis in flow. Science 2024; 383: eadj1817. 10.1126/science.adj1817 38271529

[137] Koscher BA, Canty RB, McDonald MA et al. Autonomous, multiproperty-driven molecular discovery: from predictions to measurements and back. Science 2023; 382: eadi1407. 10.1126/science.adi1407 38127734

[138] Chen J, Cross SR, Miara LJ et al. Navigating phase diagram complexity to guide robotic inorganic materials synthesis. Nat Synth 2024; 3: 606–14. 10.1038/s44160-024-00502-y

[139] Angello NH, Friday DM, Hwang C et al. Closed-loop transfer enables artificial intelligence to yield chemical knowledge. Nature 2024; 633: 351–8. 10.1038/s41586-024-07892-1 39198655

[140] Zhu D, Wang CY, Zou PC et al. Deep-learning aided atomic-scale phase segmentation toward diagnosing complex oxide cathodes for lithium-ion batteries. Nano Lett 2023; 23: 8272–9. 10.1021/acs.nanolett.3c02441 37643420

[141] Wang CY, Jing YQ, Zhu D et al. Atomic origin of chemomechanical failure of layered cathodes in all-solid-state batteries. J Am Chem Soc 2024; 146: 17712–8. 10.1021/jacs.4c02198 38874441

[142] Lin WG, Su W, Lin T et al. New insight into bulk structural degradation of high-voltage LiCoO_2_ at 4.55 V. Nano Lett 2024; 24: 7150–7. 10.1021/acs.nanolett.4c00688 38842462

[143] Li JZ, Hong YS, Yan HF et al. Probing lattice defects in crystalline battery cathode using hard X-ray nanoprobe with data-driven modeling. Energy Stor Mater 2022; 45: 647–55.

[144] Fu TY, Monaco F, Li JZ et al. Deep-learning-enabled crack detection and analysis in commercial lithium-ion battery cathodes. Adv Funct Mater 2022; 32: 2203070. 10.1002/adfm.202203070

[145] Qian GN, Zhang J, Chu SQ et al. Understanding the mesoscale degradation in nickel-rich cathode materials through machine-learning-revealed strain-redox decoupling. ACS Energy Lett 2021; 6: 687–93. 10.1021/acsenergylett.0c02699

[146] Margraf JT, Jung H, Scheurer C et al. Exploring catalytic reaction networks with machine learning. Nat Catal 2023; 6: 112–21. 10.1038/s41929-022-00896-y

[147] Han Z, Chen A, Li Z et al. Machine learning-based design of electrocatalytic materials towards high-energy lithium||sulfur batteries development. Nat Commun 2024; 15: 8433. 10.1038/s41467-024-52550-9 39505845 PMC11541723

[148] Han Z, Gao R, Wang T et al. Machine-learning-assisted design of a binary descriptor to decipher electronic and structural effects on sulfur reduction kinetics. Nat Catal 2023; 6: 1073–86. 10.1038/s41929-023-01041-z

[149] Zhai S, Xie H, Cui P et al. A combined ionic Lewis acid descriptor and machine-learning approach to prediction of efficient oxygen reduction electrodes for ceramic fuel cells. Nat Energy 2022; 7: 866–75. 10.1038/s41560-022-01098-3

[150] Ouyang B, Wang J, He T et al. Synthetic accessibility and stability rules of NASICONs. Nat Commun 2021; 12: 5752. 10.1038/s41467-021-26006-3 34599170 PMC8486869

[151] Tao SY, Sun CB, Fu SY et al. Battery cross-operation-condition lifetime prediction via interpretable feature engineering assisted adaptive machine learning. ACS Energy Lett 2023; 8: 3269–79. 10.1021/acsenergylett.3c01012

[152] Tao S, Zhang M, Zhao Z et al. Non-destructive degradation pattern decoupling for early battery trajectory prediction via physics-informed learning. Energy Environ Sci 2025; 18: 1544–59. 10.1039/D4EE03839H

[153] Zheng Z, Rong Z, Rampal N et al. A GPT-4 reticular chemist for guiding MOF discovery. Angew Chem Int Ed 2023; 62: e202311983. 10.1002/anie.202311983 37798813

[154] Zheng Z, Zhang O, Borgs C et al. ChatGPT chemistry assistant for text mining and the prediction of MOF synthesis. J Am Chem Soc 2023; 145: 18048–62. 10.1021/jacs.3c05819 37548379 PMC11073615

[155] Zuo W, Zheng H, He T et al. Large language models for batteries. Joule 2025; 9: 102037. 10.1016/j.joule.2025.102037

[156] Leng Y, Zhong Y, Gu Z et al. Intelligent, personalized scientific assistant via large language models for solid-state battery research. ACS Mater Lett 2025; 7: 1807–16. 10.1021/acsmaterialslett.4c02674

[157] Zhao S, Chen S, Zhou J et al. Potential to transform words to watts with large language models in battery research. Cell Rep Phys Sci 2024; 5: 101844. 10.1016/j.xcrp.2024.101844

[158] Dagdelen J, Dunn A, Lee S et al. Structured information extraction from scientific text with large language models. Nat Commun 2024; 15: 1418. 10.1038/s41467-024-45563-x 38360817 PMC10869356

[159] Bian C, Han X, Duan Z et al. Hybrid prompt-driven large language model for robust state-of-charge estimation of multitype Li-ion batteries. IEEE Trans Transp Electrif 2025; 11: 426–37. 10.1109/TTE.2024.3391938

[160] Zheng Z, Zhang O, Nguyen HL et al. ChatGPT research group for optimizing the crystallinity of MOFs and COFs. ACS Cent Sci 2023; 9: 2161–70. 10.1021/acscentsci.3c01087 38033801 PMC10683477

[161] Runge J, Gerhardus A, Varando G et al. Causal inference for time series. Nat Rev Earth Environ 2023; 4: 487–505. 10.1038/s43017-023-00431-y

[162] Kotobi A, Singh K, Höche D et al. Integrating explainability into graph neural network models for the prediction of X-ray absorption spectra. J Am Chem Soc 2023; 145: 22584–98. 10.1021/jacs.3c07513 37807700 PMC10591337

[163] Chen H, Yang M, Smetana B et al. Discovering electrochemistry with an electrochemistry-informed neural network (ECINN). Angew Chem Int Ed 2024; 63: e202315937. 10.1002/anie.202315937 38179808

[164] Bradford G, Lopez J, Ruza J et al. Chemistry-informed machine learning for polymer electrolyte discovery. ACS Cent Sci 2023; 9: 206–16. 10.1021/acscentsci.2c01123 36844492 PMC9951296

